# Gel-Based Approaches to Vegan Leather: Opportunities and Challenges in Mimicking Leather Properties

**DOI:** 10.3390/gels11060395

**Published:** 2025-05-27

**Authors:** Soon Mo Choi, Do Hyun Lee, Sun Mi Zo, Ankur Sood, Sung Soo Han

**Affiliations:** 1 Research Institute of Cell Culture, Yeungnam University, Gyeongsan 38541, Republic of Korea; smchoi@ynu.ac.kr (S.M.C.); sunmizo@ynu.ac.kr (S.M.Z.); 2 Korea Dyeing & Finishing Technology Institute (DYETEC), 92 Dalseocheon-ro, Seogu, Daegu 41706, Republic of Korea; lee@dyetec.or.kr; 3 School of Chemical Engineering, Yeungnam University, Gyeongsan 38541, Republic of Korea

**Keywords:** vegan leather, polymer, gels, material

## Abstract

Recently, increased global awareness of environmental sustainability and ethical consumerism has amplified the demand for sustainable alternatives to animal-derived leather. Traditional leather manufacturing faces significant ethical and ecological challenges, including greenhouse gas emissions, excessive water consumption, deforestation, and toxic chemical usage. Vegan leather has emerged as a promising solution, predominantly fabricated from petroleum-based synthetic materials such as polyurethane (PU) and polyvinyl chloride (PVC). However, these materials have sustainability limitations due to their non-biodegradability and associated environmental burdens. To overcome these issues, this review critically explores the feasibility of developing vegan leather using gel-based materials derived from natural and synthetic polymers. These materials offer precise structural controllability, excellent biodegradability, and the potential for significantly improved mechanical performance through hybridization and nanocomposite strategies. Despite their promising attributes, gel-based materials face significant limitations, including insufficient tensile strength, poor abrasion resistance, susceptibility to swelling, limited long-term stability, and challenges in scaling up for industrial production. This paper outlines the structural and physical properties required for viable leather substitutes, reviews opportunities provided by gel-based materials, addresses associated technical challenges, and proposes comprehensive strategies for enhancing mechanical properties and developing sustainable, eco-friendly production processes. Future research directions emphasize hybrid composite development, nanoparticle integration, circular manufacturing processes, and multi-disciplinary collaboration to establish gel-based vegan leather as a viable, sustainable, and market-competitive alternative to conventional animal leather.

## 1. Introduction

In recent years, global awareness of environmental protection and sustainable development has significantly increased, raising critical demands for the development of novel materials capable of overcoming environmental and ethical limitations associated with traditional industrial practices [1,2,3]. Such heightened awareness is driven by various factors, including growing recognition of climate change, concerns over resource depletion, and shifting consumer values toward ethical consumption [4,5]. Increasingly, consumers prioritize environmental sustainability and ethical considerations in their purchasing decisions, leading to a transformative shift in production processes and material selection across global industries [6]. A representative example of these transformative changes is evident in the leather industry. Animal-derived leather has been widely used as a premium material in clothing, footwear, accessories, furniture, and automotive interiors for a long time. However, conventional leather production processes entail severe environmental and ethical challenges [7,8,9]. Firstly, as a byproduct of livestock farming, leather production significantly contributes to greenhouse gas emissions, particularly methane and carbon dioxide, and requires extensive water resources while also promoting deforestation and biodiversity loss due to land expansion for animal grazing [10,11]. Additionally, the tanning process in leather manufacturing, which utilizes heavy metals such as chromium salts and various toxic chemicals, poses serious risks of water and soil contamination [12,13]. Concurrently, rapidly growing social awareness regarding animal welfare has brought ethical issues surrounding animal-derived materials into sharper focus. Increasing societal opposition to animal exploitation has reinforced consumer demands for the development and use of ethically acceptable materials [14,15,16].

In response to these issues, the concept of artificial leather or synthetic leather came into existence. The most commonly used synthetic leather materials in current markets are petroleum-based synthetic polymers, such as polyurethane (PU) and polyvinyl chloride (PVC) [17]. Although these synthetic materials offer economic competitiveness and ease of processing, they carry significant environmental drawbacks, including substantial energy consumption during production and negative environmental impacts associated with disposal. Specifically, their low biodegradability exacerbates environmental problems associated with landfilling or incineration, drawing significant criticism from a sustainability perspective [18].

As a recent innovation, “vegan leather” surfaced as an alternative material that replicates the properties and functions of conventional leather without using animal-derived components [19,20]. Vegan leather broadly encompasses leather substitutes made from plant-based or synthetic materials entirely devoid of animal products [21,22]. Therefore, to achieve genuine sustainability for vegan leather, the development of eco-friendly, biodegradable materials based on natural or synthetic polymers is essential [22]. In particular, various bio-derived polymeric materials recently formulated as gels have emerged as innovative alternatives, offering characteristics analogous to natural leather. Gel-based materials possess unique three-dimensional network structures capable of stably encapsulating liquids, allowing precise control over tactile properties, moisture retention, flexibility, and structural characteristics [23]. Natural polymer-based gels, such as gelatin, alginate, chitosan, and cellulose, offer excellent biodegradability and environmentally friendly production methods alongside considerable potential for functionality and material property enhancement, making them attractive research candidates [24,25,26]. Additionally, synthetic polymer gels, including polyvinyl alcohol (PVA), polyurethane (PU), and polyacrylamide (PAAm), exhibit superior mechanical strength and durability, thereby holding significant promise for effectively replicating the physical properties of leather.

However, to date, research specifically addressing gel-based vegan leather remains largely unexplored, with the existing literature lacking detailed analyses or comprehensive approaches. Thus, in this review, we propose, for the first time, gel-based materials derived from natural and synthetic polymers as viable vegan leather alternatives. This review systematically analyzes the properties required for effective leather substitutes and comprehensively explores strategic approaches to enhance their material characteristics. We rigorously analyze how effectively gel materials can mimic the structural and physical attributes of natural leather, present strategies to enhance their mechanical properties, and suggest directions for future research aimed at maximizing environmental sustainability. Ultimately, this work aims to provide concrete information on the advantages of gel-based materials as substitutes for leather production over other substitutes for animal-free leather production, which significantly contribute to advancements and industrial applications within this emerging research field.

## 2. Requirements for Leather Alternatives

**Figure 1:**
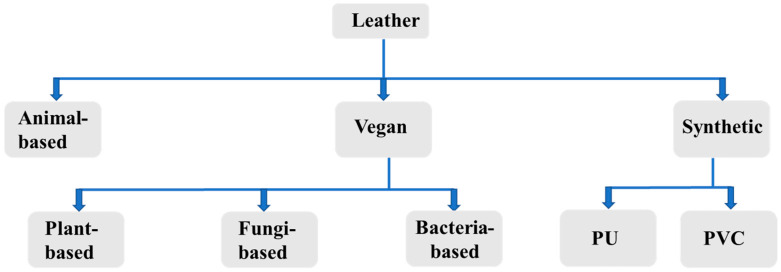
Classification of leather based on the source of development.

Throughout history, natural leather has been highly valued and extensively utilized as a premium material in various applications, including clothing, footwear, accessories, and a range of consumer products [27]. The widespread preference for natural leather is attributable to its excellent durability, tactile quality, aesthetic appearance, and overall functionality [28,29]. However, recent global trends emphasizing sustainable development and ethical consumption have heightened the urgency for developing environmentally friendly and ethically responsible alternatives capable of replacing traditional animal-derived materials. Traditional leather manufacturing processes have come under increased scrutiny due to significant environmental concerns, including substantial greenhouse gas emissions from livestock farming, excessive water consumption, deforestation resulting from pasture expansion, and contamination arising from heavy metals and toxic chemicals utilized during tanning procedures [30,31]. In response to these environmental and ethical challenges, vegan leather has emerged as a promising alternative and has been gaining considerable attention in the market. Vegan leather broadly refers to leather substitutes produced entirely without animal-derived components, typically utilizing plant-based or natural materials. Vegan leather is slightly different from artificial leather, as the latter employs materials that are petroleum-derived synthetic polymers such as polyurethane (PU) and polyvinyl chloride (PVC). Although these synthetic alternatives offer advantages such as cost-effectiveness and ease of processing, their sustainability is limited due to significant environmental drawbacks. These materials are predominantly non-biodegradable and generate considerable ecological burdens during their production and disposal phases, thereby contributing to persistent environmental issues [32,33]. Consequently, the development of biodegradable, environmentally sustainable gel-based materials derived from nature-inspired materials has been proposed as an innovative and viable alternative, thereby necessitating comprehensive and systematic research and development. Figure 1 represents the classification of vegan leather.

A fundamental requirement in developing effective leather substitutes involves a thorough understanding and precise replication of the structural and physical properties inherent to natural leather. Natural leather exhibits a highly complex structure characterized primarily by multi-layered networks of collagen fibers. These collagen fibers typically range between 2–8 µm in diameter and form intricate, densely interwoven networks, significantly contributing to the exceptional tensile strength (approximately 10–40 MPa), toughness, and overall resilience to external mechanical stresses of leather [7,34,35]. Furthermore, the porous microstructure within natural leather, comprising pore sizes ranging from approximately 10 to 300 µm, is crucial for maintaining excellent water vapor permeability (1.5–16.7 mg/cm^2^ h) and breathability, effectively regulating moisture and humidity during direct contact with human skin, thereby ensuring comfort [36,37]. The physical characteristics of leather also significantly influence consumer satisfaction and product perception. Key physical properties such as tensile strength (10–25 MPa), elongation at break (30–80%), and abrasion resistance substantially determine the material’s durability and longevity under repetitive mechanical stress conditions [19,38,39,40,41]. Moreover, sensory attributes, including tactile softness, visual appearance, and color stability, critically influence consumers’ perception of leather quality. Natural leather provides consumers with a distinctive, luxurious tactile experience and maintains uniform color stability, substantially enhancing user satisfaction. Furthermore, natural leather exhibits moisture absorption capacities ranging from approximately 7% to 12% [42], which significantly contribute to maintaining user comfort through effective moisture and humidity regulation. To successfully replicate these intricate properties of natural leather, alternative materials must precisely emulate the multi-layered collagen fiber networks and microporous structures. Specifically, leather substitutes must exhibit tensile strengths of at least 10–25 MPa, elongation at break exceeding 40%, and robust abrasion resistance [19,35]. Simultaneously, these materials must meet or exceed consumer expectations concerning tactile sensation, aesthetic appearance, and color stability. From the standpoint of environmental sustainability, novel materials should substantially reduce greenhouse gas emissions and water usage during production by at least 50% compared to conventional leather manufacturing processes and ensure full biodegradability upon disposal to minimize ecological impact [43]. Finally, from an economic perspective, alternative materials must achieve at least a 20–30% cost reduction relative to traditional leather through efficient, scalable production processes to ensure commercial viability [44]. Only when these comprehensive requirements are adequately satisfied can truly sustainable and competitively viable alternatives to natural leather be successfully developed and commercialized. Table 1 summarizes the salient features of natural leather that have to be reflected by their sustainable alternative.

## 3. Alternatives to Animal-Based Leather

The animal-based leather industry causes serious damage to the environment while consuming a substantial amount of resources. Thanks to the modern-day technological advancements in the field of material sciences, there are many sources that could be explored for leather production while minimizing the damage caused due to environmental hazards. Some of the sources explored for leather production are plant-based, fungi-based, and bacterial-based natural leather substitutes.

### 3.1. Plant-Based Leather

**Figure 2:**
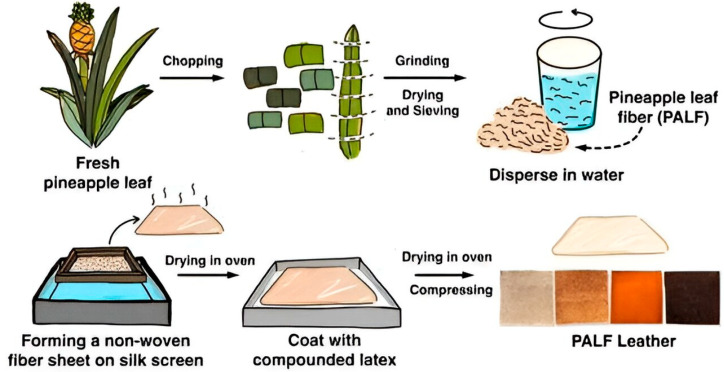
Scheme representing the formation of PALF leather [31].

Nature has always inspired and nourished mankind to develop innovative approaches in creating novel materials with remarkable properties. Plant-derived cellulose has been used in the development of leather. The fibrous architecture of cellulose has been explored to devise a substitute for animal leather [45]. The production of leather using cellulose is remarkably dependent upon the source of cellulose. Piatex^®^ (São Paulo, Brazil), as a global leader in plant-based bio-leather production, has created leather from the fibers of pineapple leaves, a byproduct of the pineapple industry. A similar study has also been reported by Duangsuwan et al., where a leather alternative was developed using pineapple leaf fiber (PALF) and natural rubber (Figure 2) [31]. Moreover, Desserto^®^ (Jal, Mexico), another plant-based leather-making company, has used cactus leaves to develop bio leather [7]. A key aspect in plant-based leather is its satisfactory mechanical features, but the cost of production could sometimes increase as binders are to be included, as on the choice of plant source. Also, batch-to-batch variation and less durability could be two key aspects that have to be addressed for the industrial viability of plant-based leather.

### 3.2. Fungi-Based Leather

**Figure 3:**
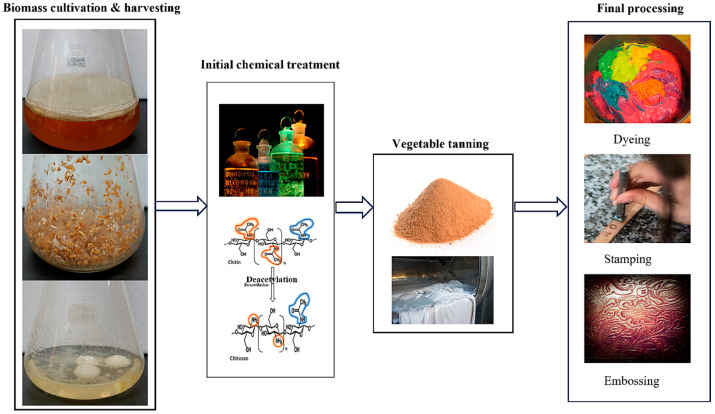
Overview of the processing of the substitute for fabricating a fungal-based Leather [46].

Recently, fungal biomass has been explored for diverse applications, including adhesive coatings, wood dressings, food wrapping, developing membranes for filtration, and biopolymer sheets from which textiles and leather substitutes can be derived [46,47]. An important aspect of the utility of fungal biomass in all these applications is the fibrous nature of fungal mycelium. The physicochemical attributes, mechanical features, and thermal behavior of fungal-based biopolymers were found to be near those of conventional polymers, thus making them an ideal candidate for the leather industry [48]. It has also been reported that the mechanical and physicochemical features of the fungal-based materials could be altered by changing and optimizing their feed substrates [49]. Another important aspect that makes fungal-based material an ideal candidate for the leather industry is its growth requirements, which depend on highly inexpensive substrates, thus reducing the cost of production to a substantial level. It is also reported that the carbon footprint of the fungal leather substitute is reported to be 2.76 kg CO_2_-eq per m^2^t, which is 1250% less than bovine leather [50]. A detailed overview representing the overall processing of fungal leather substitutes is presented in Figure 3. A summary of different fungal sources used in the leather industry with their key attributes is presented in Table 2.

### 3.3. Bacterial Cellulose (BC)-Based Leather

BC is an alternative to plant cellulose and is produced by some bacteria with a highly hydrophilic nature, more biocompatibility, malleability, robustness (10 times stronger than plant-derived cellulose), and is highly hydrophilic [54,55]. A substantial increase in the utility of BC has been observed across many industries, including wound dressings [56,57], acoustic products [58], and the textile industry [59]. Showcasing the BC as a potent substitute in the leather industry is a result of the production of membrane fibers by the genus *Komagataeibacter*, precisely, the strain of *K. xylinum* [60]. This bacterial strain can produce cellulose pellicles, and, upon drying, it demonstrates a leather-like material. It is estimated that by the end of 2030, the carbon footprint of the fashion industry alone will increase to 2791 tons [54]. These numbers could be significantly improved by utilizing alternatives to natural leather. A major advantage in producing BC-based leather is the upscaling process, which employs a simple fermentation process, thus making the approach economically viable. Another key aspect that makes BC a unique candidate for leather production is its minimal batch-to-batch variation, which supports improving quality. A major disadvantage that is associated with BC as a substitute for the leather industry is the requirement of specific conditions, which sometimes could add to the overall cost of production. Also, the fungal and bacterial-based alternatives to leather could sometimes be less scratch-resistant and water repellent. This allows for more alternatives that could be explored in the leather development. A comparative analysis of different leather substitutes is presented in Table 3.

## 4. Opportunities for Gel-Based Materials as Leather Alternatives

Gel materials, characterized by their unique structure where liquids are immobilized within a three-dimensional polymeric network, exhibit intermediate properties between solids and liquids. Recently, gels have drawn significant attention across various fields, such as biomedical applications, drug delivery systems, tissue engineering, and functional materials, owing to their unique and versatile characteristics [61,62,63,64]. In particular, gels derived from natural and synthetic polymers have shown substantial potential for effectively replicating the structural, physical, and functional properties inherent in natural leather [65].

One of the primary advantages of gel-based materials lies in their precise controllability of structural characteristics. Natural leather features a complex, multi-layered structure composed primarily of collagen fibers, requiring a highly sophisticated microstructural design for effective mimicry. The polymer chain cross-linking densities in gel materials can be precisely adjusted at the molecular level, or advanced fabrication techniques such as freeze-drying, three-dimensional (3D) printing, and self-assembly can be employed to replicate intricate fibrous and multi-layered structures resembling collagen networks. This structural similarity is crucial for reproducing leather’s excellent tensile strength, toughness, and shape recovery characteristics, enabling gels to exhibit comparable mechanical resilience under external stresses. Another notable advantage of gel-based materials is their capability for precise modulation of porous structures. Recent studies suggest that gel materials can achieve comparable porous structures through meticulous control of pore size and distribution, potentially replicating the superior breathability and moisture permeability of natural leather [66,67]. Advanced processing methods such as freeze-drying and emulsion templating can further refine these internal structures, representing essential strategies for developing gel-based vegan leather [68]. Additionally, gel materials offer significant versatility through their ease of incorporating diverse functional additives. For instance, embedding antibacterial agents, such as silver nanoparticles or chitosan, within the gel matrix can effectively prevent microbial-induced degradation and odor issues during prolonged use [69,70,71,72]. Similarly, introducing ultraviolet (UV)-resistant materials, such as titanium dioxide (TiO_2_) nanoparticles, can substantially mitigate color fading and physical deterioration typically experienced by leather under extended exposure to sunlight [73,74]. Recent advances in self-healing gel development demonstrate the potential for autonomous repair of minor scratches and damages, significantly enhancing material durability. Furthermore, dyes and various organic or inorganic fillers can be readily incorporated into gels, offering excellent adaptability in visual aesthetics and coloration [75].

**Figure 4:**
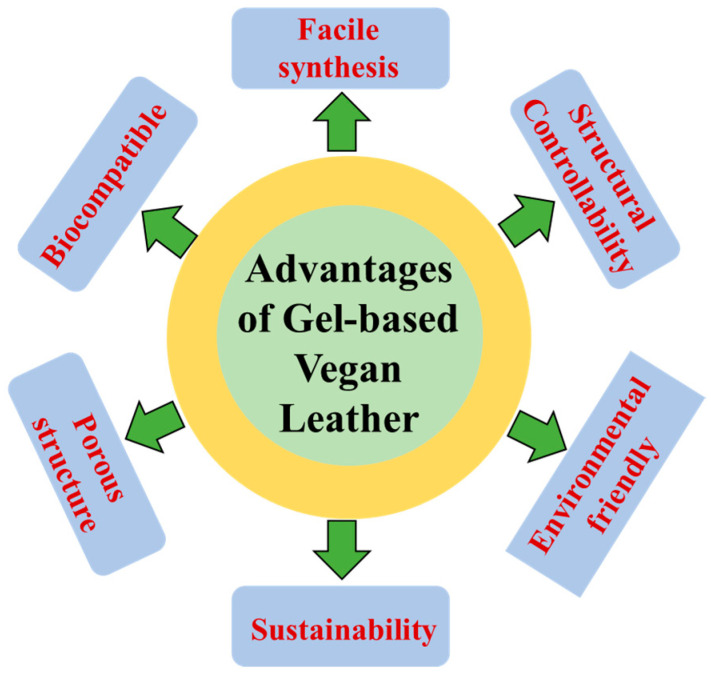
Advantages of gel-based vegan leather.

From a cleaner environmental perspective, gel-based materials offer considerable ecological benefits. Natural polymer-derived gels such as gelatin, alginate, chitosan, and cellulose exhibit remarkable biodegradability and biocompatibility, significantly reducing environmental hazards throughout their production and disposal stages. These materials eliminate the need for toxic chemicals or organic solvents typically used in traditional chromium tanning processes, thereby enabling environmentally friendly aqueous-based manufacturing methods. Consequently, gel-based materials significantly reduce carbon footprints and fully support circular economy principles through recyclability and minimal ecological disruption upon disposal. The overall advantage of gel-based vegan leather is presented in Figure 4.

Hybridization and composite formulation strategies have emerged as critical methodologies for enhancing the mechanical performance of gel-based materials. Recent research highlights that incorporating nanoparticles, including cellulose nanocrystals (CNC) [76,77], graphene oxide (GO) [78,79], and carbon nanotubes (CNT) [80,81], into gel matrices significantly improves their tensile strength and durability. Such composite strategies leverage the high surface area and outstanding mechanical properties of nanoparticles, facilitating efficient stress transfer within gel networks and enhancing structural reinforcement.

In conclusion, gel-based materials as a substitute for natural leather possess considerable potential due to their structural precision, multifunctionality, and outstanding environmentally friendly approach. With further intensive research and development addressing current technical and economic challenges, gel materials are expected to play a pivotal role in transitioning toward a safe environment and an ethically responsible leather industry.

## 5. Challenges and Limitations of Gel-Based Materials for Leather Alternatives

For gel-based materials to become a practical and commercially viable alternative to natural leather, several significant technical challenges and limitations must be systematically addressed and overcome. The first major challenge pertains to the insufficient mechanical properties of gel-based materials. Although gels typically exhibit desirable softness and flexibility, their mechanical characteristics, specifically tensile strength and abrasion resistance, are considerably inferior to those of natural leather. According to research, the tensile strength of most gel-based materials is typically much lower [82,83] than the characteristic tensile strength of conventional leather. This inherent mechanical weakness restricts the durability and practical applicability of gels under prolonged and repetitive mechanical stresses encountered in daily use. To overcome these limitations, extensive research is required to reinforce gel networks through incorporating high-strength fillers and nanoparticles (e.g., cellulose nanocrystals (CNC), graphene oxide (GO), and carbon nanotubes (CNT)) or through strategic polymer hybridization. Recent studies indicate that such composite strategies can significantly enhance the mechanical strength and performance of gel-based materials [84,85,86]. The second critical limitation involves poor water and humidity resistance exhibited by most gel materials. Due to their inherent porous structures, gels readily absorb moisture, causing swelling that severely compromises structural stability and original physical properties. This swelling phenomenon can lead to significant shape deformation and functional degradation, especially under prolonged exposure to humid or wet environments. Therefore, addressing this limitation through advanced cross-linking technologies and hydrophobic surface treatments is essential. Current research has intensified in developing advanced surface engineering approaches, including surface modification and incorporating chemically reactive functional groups, to effectively mitigate swelling and enhance water and humidity resistance in gel materials [87,88,89,90]. A third significant challenge is ensuring the long-term stability of gel-based materials. Over extended periods, polymeric networks within gel materials can gradually degrade or undergo structural alterations, substantially deteriorating their physical and chemical properties. Such time-dependent degradation significantly reduces the reliability and usability of gel materials, especially for long-term applications. Extensive research into enhancing durability and stabilizing polymeric networks is, thus, critically important. Potential strategies include incorporating antioxidants, ultraviolet (UV) stabilizers, or self-healing polymer technologies to slow degradation processes and significantly enhance long-term durability and stability. Lastly, scalability and economic feasibility in gel-based material production present significant barriers to their commercial viability. The manufacturing processes involved in gel production often require complex procedures, substantial processing time, and high costs, making large-scale production economically challenging. Achieving precise control over pore sizes and structural intricacies typically demands additional processing time and costs, further limiting economic feasibility for mass production. Therefore, to enable effective commercialization, significant advances in manufacturing process simplification, automation, and the development of cost-effective, environmentally friendly large-scale production methods are urgently needed. Additionally, developing resource-efficient technologies, such as recycling and reusing raw materials, is imperative to maximizing resource utilization efficiency and reducing the overall production costs.

While efforts are being made to include gel-based leather alternatives, it is very crucial to study the behavior and performance of gel-based and other synthetic substitutes in real-use conditions. Natural leather, upon prolonged exposure to the sun, develops cracking, peeling, discoloration, and a fading-like appearance. In case of non-animal substitutes for leather, this would be a persistent problem, as these leather substitutes will be more prone to damage. Inclusion of heat-resistant nanomaterials and biomolecules as fillers in the network of these leather substitutes could be highly effective. Moreover, depending on the composition of the non-animal leather substitutes, different behavior to friction is expected. Synthetic leather made of PVC or PU could imitate real leather in terms of durability and friction properties, but these characteristics have to be carefully regulated in case of plant-based, fungi-based, BC-based, or gel-based leather substitutes. The type of additives would greatly influence many properties of non-animal leather substitutes and would indeed direct the development of an environmentally benign leather.

**Figure 5:**
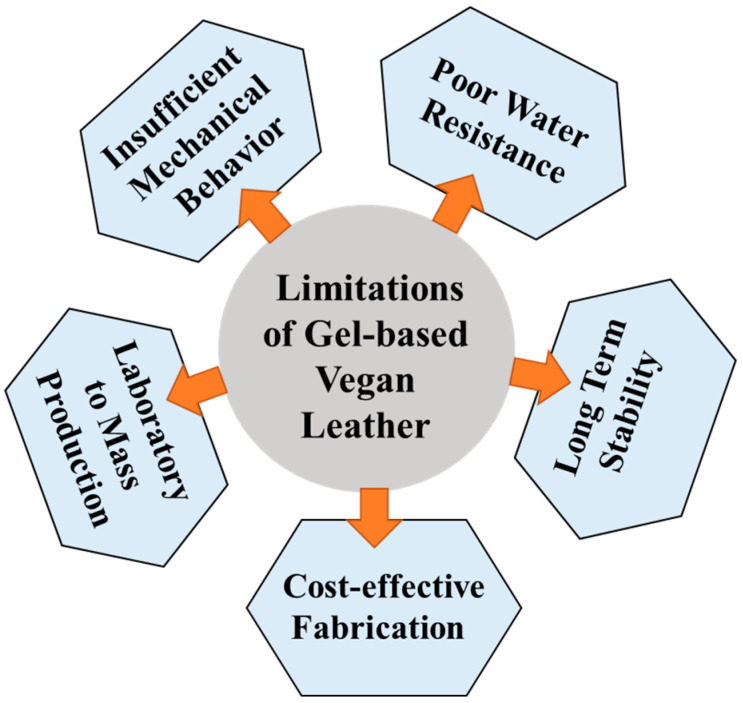
Challenges and limitations of gel-based vegan leather.

In conclusion, for gel-based vegan leather to effectively substitute natural leather and achieve industrial applicability, it is crucial to systematically overcome multiple technical and economic challenges, including enhancing mechanical properties, improving water and humidity resistance, ensuring long-term stability, and achieving cost-effective, scalable production processes. Multidisciplinary collaboration and ongoing research and development are essential to addressing these challenges. Through the advancement of relevant technologies, gel-based materials hold significant promise for substantially advancing sustainable and ethical leather industries. The summary of the challenges and limitations in developing gel-based vegan leather is presented in Figure 5.

## 6. Future Strategies for the Development of Gel-Based Vegan Leather

For gel-based substitutes to successfully replace natural leather and establish market competitiveness, strategic approaches must systematically address current technical limitations while simultaneously maximizing an eco-friendly approach. Future research and development should primarily focus on two crucial strategies: “Hybridization and composite material technologies for mechanical performance enhancement” and “Establishing an eco-friendly manufacturing process”. Firstly, advancing material manufacturing technologies is critical to overcoming the inherently limited mechanical properties of gel-based materials. While gels naturally exhibit desirable softness and tactile qualities, they typically fall short in terms of tensile strength, abrasion resistance, and overall durability compared to natural leather. To mitigate these deficiencies, research should actively pursue composite strategies involving high-performance nanoparticles and various fillers. Judiciously selected nanoparticles can provide superior mechanical strength and large surface areas, effectively enhancing stress transfer and structural reinforcement within the gel matrix. In particular, nanocellulose has attracted significant attention as a promising additive due to its exceptional biodegradability, biocompatibility, and mechanical performance. Further research into nanoparticle–gel interactions and dispersion stability is essential for optimizing the durability and functionality of gel-based materials. Moreover, the polymer composite fabrication approach between natural and synthetic gels represents another crucial research area. By combining or layering natural polymer-based gels with synthetic polymer-based gels, it is possible to achieve the desired outcome in terms of acceptability and durability. Recent advances in fabrication techniques, including 3D printing and laminated fabrication methods, enable precise structural control, significantly enhancing the functional properties and structural accuracy of hybrid materials. Additionally, utilizing artificial intelligence (AI)-based predictive models during material design could further optimize interactions among polymers and fillers, facilitating improvements in mechanical properties and long-term durability. Secondly, establishing eco-friendly manufacturing processes is essential to maximize the ecological advantages of gel-based vegan leather. Although gels are generally evaluated as eco-friendly and biodegradable, improvements in manufacturing processes are needed to minimize the use of harmful chemicals, solvents, and energy consumption. Emphasis should be placed on adopting aqueous-based processing methods and selecting biodegradable raw materials from the outset, alongside the use of less toxic and environmentally safe crosslinkers. Furthermore, introducing circular manufacturing practices that maximize resource efficiency through recycling and reuse of waste and byproducts is critical. For example, recovering and reusing unused gel material or byproducts generated during the manufacturing process, as well as recycling water and energy within production cycles, would significantly enhance resource utilization efficiency. Concurrently, developing fully biodegradable designs that minimize environmental impact throughout the entire product lifecycle should also be prioritized, ensuring that materials naturally decompose post-use without adverse ecological effects.

To systematically develop these core strategies, interdisciplinary collaboration and multidisciplinary approaches are indispensable. Experts from various fields, including materials science, polymer engineering, nanotechnology, biotechnology, and environmental engineering, must collaborate to facilitate comprehensive material design and manufacturing process innovation. Additionally, strengthening industry–academia partnerships is vital for rapidly commercializing research outcomes and building robust infrastructures that support sustainable industry growth. Establishing international standards and criteria for evaluating the performance of gel-based materials through rigorous verification and validation studies is also necessary to ensure technical reliability and market readiness. Further, in order to reduce the cost of the overall product, bulk manufacturing of gels could also be implemented, powered by automation. This will streamline the process of gel-based leather making.

For gel-based leather substitutes, continuous advancement in hybridization and composite technologies to improve mechanical behavior, along with establishing eco-friendly and circular manufacturing processes, is essential. Through integrated and systematic approaches, gel-based vegan leather is expected to become commercially successful and significantly contribute to sustainable and ethically responsible leather industries.

## 7. Conclusions

This review comprehensively assessed the potential of gel-based materials as alternatives to traditional animal-derived leather, critically discussing both the opportunities and limitations associated with gel-based vegan leather. Gel-based materials demonstrate considerable potential for mimicking the physical and structural characteristics of natural leather due to their distinctive three-dimensional polymeric network structure. Notably, their ability to precisely control porous structures allows them to replicate the superior moisture permeability and breathability of natural leather. Furthermore, composite strategies incorporating nanoparticles and high-performance fillers significantly enhance mechanical strength and abrasion resistance, addressing critical performance shortcomings.

From the perspectives of environmentally safe and ethical responsibility, gel-based materials can exert substantial positive impacts on the leather industry. Bio-based gels derived from natural raw materials offer significant biodegradability, substantially reducing environmental burdens upon disposal. Additionally, implementing eco-friendly manufacturing processes that minimize the use of toxic chemicals and reduce greenhouse gas emissions will further contribute significantly to the sustainable transformation of the leather industry. Moreover, integrating circular manufacturing practices—emphasizing waste reuse and recycling—can enhance resource efficiency and establish environmentally friendly industrial ecosystems. Despite their promising advantages, several technical and economic limitations must be overcome to successfully commercialize gel-based materials. Addressing insufficient mechanical properties remains a major technical challenge, alongside the need for further research into long-term structural stability and durability under prolonged use conditions. Therefore, meticulous design and stabilization techniques of polymeric networks are essential to maintain consistent performance. Furthermore, optimizing and simplifying manufacturing processes to achieve economic feasibility and scalability are critical steps toward effective commercialization. Future research should focus on developing hybrid materials that balance structural integrity and functionality, extensively incorporating advanced nanoparticles and sustainable raw materials. Evaluating long-term stability and conducting validation studies under realistic conditions are crucial to establishing technological reliability. Additionally, active interdisciplinary collaboration and industry–academic partnerships are indispensable to further enhance the performance of gel-based vegan leather and realize material properties equivalent to or surpassing traditional leather. Further, the acceptance of gel-based substitutes for natural leather among customers is a crucial factor. The perspective of society has to be considered, as for some customers, appearance is more important, whereas for some, environmental protection is a key issue.

Gel-based vegan leather holds significant promise as an innovative and sustainable alternative material for the future. Nevertheless, sustained and systematic research efforts are essential to address current technical limitations and optimize production processes, thereby achieving genuine commercial viability and environmental sustainability. Through comprehensive and continuous research endeavors, gel-based materials are expected to play a pivotal role in driving the sustainable transition of the leather industry.

## Figures and Tables

**Table 1:** Salient features of natural leather.

Property	Range	Reference
Diameter of collagen fibers	2–8 µm	[34]
Tensile strength	10–40 MPa	[35]
Pore size	10–300 µm	[36]
Water vapor permeability	1.5–16.7 mg/cm^2^ h	[37]
Elongation at break	30–80%	[19]
Moisture absorption capacities	7–12%	[42]

**Table 2:** Comparison of parameters of fungal-based leather obtained from different fungal species.

Fungal Species	Young’s Modulus	Strain at Break (%)	Tensile Strength (MPa)	Ref.
*Agaricus bisporus*	7 GPa	-	100–200	[51]
*Fomes fomentarius*	22.21 ± 3.38 kPa	-	0.51 ± 0.12	[52]
*Ganoderma lucidum*	-	26	0.392	[53]
*Phellinus ellipsoideus*	-	101	1.2	[49]

**Table 3:** Comparison of different aspects among various leather substitutes.

	Animal Leather	Synthetic Leather	Fungi-Based Leather	Bacterial Cellulose-Based Leather	Plant-Based Leather
Biodegradability	Limited	Limited	Highly	Highly	Highly
Cost of production	High	Moderate	Moderate	Moderate	Less
Mechanical attributes	Good	Moderate	Less	Less	Less
Environmental impact	High	Moderate	Less	Less	Less
Advantage	Durable	Ease in bulk production	Environmentally friendly	Ease in bulk production	Economically viable
Disadvantage	Environmental hazard	Batch-to-batch variations	Species-dependent processing	Requirement of specific conditions	Less yield

## Data Availability

No new data were generated.
